# Microbiome-Metabolome Analysis Insight into the Effects of the Extract of *Phyllanthus emblica* L. on High-Fat Diet-Induced Hyperlipidemia

**DOI:** 10.3390/metabo14050257

**Published:** 2024-04-29

**Authors:** Jiahao Wang, Jijing Dong, Furong Zhong, Sha Wu, Guangqin An, Wan Liao, Luming Qi, Yuntong Ma

**Affiliations:** 1 State Key Laboratory of Characteristic Chinese Medicine Resources in Southwest China, Chengdu University of Traditional Chinese Medicine, Chengdu 611137, China; 2 School of Pharmacy, Chengdu University of Traditional Chinese Medicine, Chengdu 611137, China; 3 School of Health Preservation and Rehabilitation, Chengdu University of Traditional Chinese Medicine, Chengdu 611137, China

**Keywords:** *Phyllanthus emblica*, hyperlipidemia, gut microbiota, metabolomics, short-chain fatty acids

## Abstract

The fruit of *Phyllanthus emblica* L. (FEPE) has a long history of use in Asian folk medicine. The main bioactive compounds in FEPE are polyphenols, known for their potent antioxidant, anti-inflammatory, and hypolipidemic activities. The present study aimed to investigate the intervention effect of FEPE (100 and 200 mg/kg) on hyperlipidemia for 8 weeks and preliminarily explored the potential mechanism by microbiome-metabolome analysis. The results showed that a high-dose FEPE (200 mg/kg) effectively alleviated dyslipidaemic symptoms and body weight gain in hyperlipidemic mice induced by a high-fat diet (HFD). Microbiome analysis showed that FEPE altered the structure of the intestinal microbiota, which included an increase in specific probiotics (such as *Akkermansia*, *Anaerovorax*, and *Bacteroides*) and a decrease in harmful bacteria (including *A2*, *Acetitomaculum*, *Candidatus_Arthromitus*, *Lachnospiraceae_NK4A136*_*group*, *Lachnospiraceae_NK4B4_group*, *Rikenella*, and *Streptococcus*), as well as a reduction in the level of short-chain fatty acids (SCFAs). In addition, significant changes in the hepatic metabolome were observed, and eight key metabolites associated with betaine metabolism, lysine degradation, methionine metabolism, and fatty acid metabolism pathways were primarily filtered. The correlated analysis identified several key “microbiota-metabolite” axes in the treatment of hyperlipidemia by FEPE extract. In conclusion, the present study is expected to provide a basis for treating hyperlipidemia with FEPE from the perspective of the microbiome-liver metabolome axis.

## 1. Introduction

Hyperlipidemia is a common chronic dyslipidemia disease accompanied by an increase in total cholesterol (TC), triglyceride (TG), or low-density lipoprotein cholesterol (LDL-c), and a decrease in high-density lipoprotein cholesterol (HDL-c) [1,2,3]. It has become a main risk factor for the development of fatty liver, atherosclerosis, and related cardiovascular diseases. As people’s lifestyles and diet habits change, the morbidity and mortality rates of this disease are increasing year on year, with a trend towards younger age. In clinical practice, statins are frequently prescribed as the primary medication for managing hyperlipidemia. Typically, these drugs are known for their favorable tolerability and safety profiles. Nevertheless, the prolonged utilization of these pharmaceuticals could potentially lead to risks of hepatotoxicity and myotoxicity [4,5,6]. Nowadays, some extracts of natural products have been recognized to regulate gut microbiota and liver metabolism, further preventing the development of hyperlipidemia. Therefore, tapping into natural products with lipid-lowering properties is a promising strategy for preventing this disease.

*Phyllanthus emblica* L. (also popularly known as amla) belongs to the family of Euphorbiaceae and is mainly distributed in tropical and subtropical regions such as India, Malaysia, and China [7,8]. The fruits of *Phyllanthus emblica* L. (FEPE) play an important role in Indian traditional medicine, have a high medicinal and nutritional value, and act as a strong Rasayana (techniques for lengthening lifespans) in Ayurveda [9]. Research studies have shown that FEPE has broad biological effects, including antioxidant, anti-inflammatory, lowering cholesterol, and liver protection, which are closely related to the rich polyphenolic components such as gallic acid, ellagic acid, and quercetin [1,10,11]. Several polyphenol-rich extracts have been shown to exert their beneficial effects regarding hyperlipidemia. Li et al. investigated that *Allium cepa* extract enriched in polyphenols could alleviate hyperlipidemia by down-regulating 3-hydroxy-3-methylglutaryl-CoA (HMG-CoA) reductase and up-regulating low-density lipoprotein receptor [12]. Similarly, Rzepecka-Stojko et al. found that polyphenol-rich ethanol extract from bee pollen was also effective in alleviating the symptoms of hyperlipidemia, especially the levels of oxidized low-density lipoproteins [13]. However, the hypolipidemic effect and related mechanism of FEPE are less well studied.

As far as we know, a long-term high-fat diet (HFD) habit is one of the risk factors that cause hyperlipidemia. HFD can remodel the profiling of gut microbiota, which was closely associated with the onset of this disease. HFD changed many key gut microbiota, such as *Akkermansia*, *Lactobacillus*, and *Bacteroides*, and there is a significant correlation between these microbiota and TC, TG, and LDL-c [1,14,15]. The metabolites of short-chain fatty acids (SCFAs) produced by the gut microbiota are also important contributors associated with the onset of hyperlipidemia [16]. As a key target organ for nutritional therapy, the liver plays a key role in maintaining lipid homeostasis. Research reports have proven that the liver is responsible for regulating a variety of pathways of lipid metabolism, including lipogenesis and cholesterol metabolism. In addition, it has also been established that changes in metabolites and metabolic pathways in the liver are critical in influencing changes in lipid levels. Modern studies have also confirmed that the liver metabolome and microbiome axes are closely associated with high-fat diet-induced hyperlipidemia [17]. Thus, the improvement of host metabolism and gut health might be considered the target of alleviating hyperlipidemia.

Given the important role of the microbiome-liver metabolome axis in hyperlipidemia pathology, the objectives of this study were to examine the anti-hyperlipidaemic effects of FEPE and to explore potential mechanisms using a microbial-metabolomic approach, which could provide new ideas about the lipid-lowering mechanism of FEPE.

## 2. Materials and Methods

### 2.1. Materials and Reagents

The dried fruits of *Phyllanthus emblica* L. were purchased from Chengdu Lotus Pond Herb Market and identified by Professor Yun-tong Ma, who is an expert on medicinal plants at Chengdu University of Traditional Chinese Medicine (Chengdu, China). Normal diet (10% calories from fat, D12450B) and HFD (45% calories from fat, D12451) were purchased from Beijing SiPaiFu Company (Beijing, China) [18,19]. The specific composition of the feed is shown in Table S1.

Chemical Reference Standards of gallic acid (CHB161212), corilagin (CHB170917), chebulic acid (CHB171219), and ellagic acid (CHB160411) were purchased from Chroma Biotechnology Co., Ltd. (Chengdu, China) (All purity of reagents > 98%). Methanol (chromatographic grade) was purchased from Thermo Fisher Scientific (Shanghai, China). Deionized water used for chromatographic analysis was produced using an ultrapure water system (Millipore, Billerica, MA, USA). Other analytical grade reagents were supplied by Chron Chemicals Co., Ltd. (Chengdu, China).

### 2.2. The Components Analysis of FEPE

The sample preparation and components analysis for the FEPE samples were carried out according to the Chinese Pharmacopeia [20]. The dried fruits were crushed in a small medicine crusher and screened through an 80-mesh pharmacopeia sieve to obtain some brown powder. The powder of 0.100 g was weighed and placed in a dry conical flask, added to methanol, and extracted ultrasonically for 1 h. The methanol was added again to compensate for the lost mass and filtered through a 0.45 μm microporous membrane to obtain the test solution. The main chemical components of gallic acid, corilagin, chebulinic acid, and ellagic acid in FEPE were monitored by using our previously established method [21]. A Shimadzu system (Shimadzu, Kyoto City, Japan) equipped with an LC-20AT quaternary pump, a SIL-20A XR autosampler, a CTO-20AC column oven, and an SPD-20A UV/Vis detector was utilized to determine bioactive compounds of FEPE. An Agilent ZOR-BAX Eclipse XDB-C18 (4.6 mm × 250 mm, 5 mm) column was applied to separate objective compounds. Other HPLC conditions are listed below: column temperature: 30 °C; mobile phase: methanol (A) and 0.1% phosphoric acid (B); flow rate: 1 mL/min^−1^; elution gradient: (0~15 min, 5% A; 15–35 min, 5~37% A; 35~39 min, 37~47% A; 39~60 min, 47~60% A); injection volume: 5 μL; detection wavelength: 273 nm.

### 2.3. Animals and Treatment

Fifty specific pathogen-free (SPF)-grade male C57BL/6J mice with body weight 20 ± 2 g were purchased from Beijing SiPaiFu Experimental Animal Co., LTD., (No. SCXK 2019-0010, Beijing, China). Animals were adapted to the laboratory environment with a relative humidity of 60 ± 5%, a standard 12 h light/dark cycle, and a temperature of 22 ± 2 °C for 7 days before the experiment. The animals were allowed free access to standard chow and sterilized water. All procedures involving the handling of animals were conducted by the Chengdu University of Traditional Chinese Medicine Guide for the Care and Use of Laboratory Animals and were approved by the Institutional Ethics Committee of the Chengdu University of Traditional Chinese Medicine (Protocol number 2020-36).

The FEPE water extract was obtained according to the standard TCM decoction method. All the dried fruit powder was decocted for 2 h with an addition of 10 times the volume of distilled water (1:10, *w*/*v*) and then filtered. The residue was boiled again for 30 min with an addition of 10 times the volume of distilled water (1:10, *w*/*v*). The extracted filtrates were combined and concentrated into 100 mg/kg and 200 mg/kg crude drugs. The concentrated decoction was sealed and placed at 4 °C for later use.

The mice were randomly divided into 2 groups: one group of mice was fed a normal diet (*n* = 10), and the other group of mice was fed a high-fat diet to induce hyperlipidemia (*n* = 40). After 8 weeks, the TC and TG levels of each mouse were measured respectively. From the normal diet group of mice, the six mice were selected as normal control group mice (NC, *n* = 6) based on the levels of TC, TG, and body weight. From the mice in the HFD group, twenty-four mice were selected and randomly divided into four groups (*n* = 6) based on significantly increased levels of TC, TG, and body weight compared to the NC group. During the treatment, the mice were given the FEPE_L (100 mg/kg/day), FEPE_H (200 mg/kg/day), and Rosuvastatin Calcium Tablets (RCT, 0.1 mg/kg) or saline orally for 8 weeks according to a previous study and Chinese pharmacopeia [22]. FEPE_L (100 mg/kg/day) and FEPE_H (200 mg/kg/day) were equivalent to 75 mg/kg/d and 150 mg/kg/d in humans, respectively.

**Figure 1:**
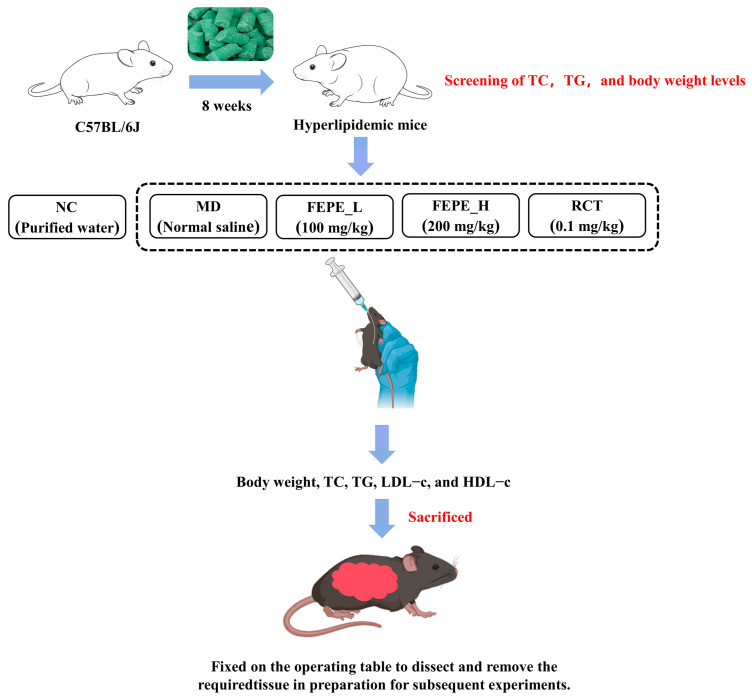
Animal experimental protocol and design.

All mice were gavaged at the same time. The time of gavage administration was eight weeks for each group. The experimental design of the grouping and intervention are displayed in Figure 1.

### 2.4. Biochemical Analysis

The blood samples were placed in EP tubes containing sodium heparin after being taken from the eyelids of mice and then centrifuged for 15 min at 3500 rpm to obtain plasma. The plasma levels of TG, TC, HDL-c, and LDL-c were measured by using the Mindray BS-200 automatic biochemical analyzer with the corresponding commercial kits (Mindray, Shenzhen, China).

### 2.5. Histopathological Analysis

The liver tissue was fixed in 4% paraformaldehyde, dehydrated using an alcohol gradient, and embedded in paraffin. Sections 3 μm thick were cut and stained with hematoxylin and eosin (H&E) for pathological analysis. The stained sections were viewed using a light microscope with a magnification of 20.0 (Leica, DM500, Wetzlar, Germany); the LIOO imaging system was used.

### 2.6. Gut Microbiota Analysis

The Genomic DNA was extracted from the fecal samples using a DNA Kit (Cwbio, Taizhou, China) according to the manufacturer’s protocols. The quality and concentration of DNA were determined by 1.0% agarose gel electrophoresis and a NanoDrop^®^ ND-2000 spectrophotometer (Thermo Scientific Inc., Waltham, MA, USA) and kept at −80 °C before further use. The universal primers 338F/806R (338F 5′-ACTCCTACGGGAGGCAGCAG-3′, 806R 5′-GGACTACGVGGGTWTCTAAT-3′) were used to amplify the V3–V4 region of the gut bacteria’s 16S rDNA by PCR.

The samples were sent to Shanghai Magi Biomedical Technology Co., LTD. (Shanghai, China) to perform the requested measurements. Data analysis was performed in the United States using biological cloud platforms (https://login.majorbio.com/login, accessed on 16 December 2022). ANOSIM analysis was used to analyze the similarity between groups, and LEfSe analysis was used to analyze the genus-level differences between NC, MD, and FEPE groups (LDA score > 3, *p* < 0.05).

### 2.7. SCFAs Analysis in Feces

The SCFAs in fecal samples were extracted and determined according to the method of our reference with appropriate modifications [16,23]. Briefly, the feces were suspended in 1 mL of ultrapure water to obtain a 20% (*m*:*v*) slurry. After homogenization and centrifugation, the supernatant of each sample was mixed with metaphosphoric acid (25%) at a ratio of 5:1 (*v*:*v*). After shaking for 5 min, the mixtures were centrifuged at 4 °C (12,000× *g*, 15 min), and the supernatant was collected. 

The supernatant liquid was analyzed by the Agilent 7820A gas chromatograph (Agilent Technologies, Inc., Santa Clara, CA, USA) equipped with a DB-FFAP column (30 m × 250 μm × 0.25 μm) according to the following program: the initial temperature was 60 °C (1 min) and raised to 120 °C (30 °C/min), increased to 170 °C (5 °C/min), raised to 220 °C (15 °C/min, maintained 6 min), injection volume 1 µL. The temperatures of the injector and detector were 250 °C and 270 °C, respectively. Standard curves were constructed, and the SCFAs levels were calculated.

### 2.8. Liver Metabonomics Analysis

A sample of 80 mg of liver tissue was weighed, mixed in pre-cooled methanol/water (1:1300 µL), and centrifuged. The supernatant was then transferred to a 1.5 mL EP tube. The precipitate was extracted again in the same way; the supernatants of the two extracts were mixed, and the mixed supernatant was blown dry under nitrogen. The blown dry extract was re-dissolved in 300 µL of methanol-water (4:1), vortexed for 5 min, and centrifuged at 13,000 r/min^−1^ at 4 °C for 15 min before being analyzed. The supernatant was collected and filtered through a 0.22 μm filter membrane for the next analysis.

The ultra-high-performance liquid chromatography coupled with mass spectrometry/mass spectrometry (UPLC-MS/MS) was used to analyze the metabolites of liver samples (Thermo Fisher Scientific, Waltham, MA, USA). Accucore™ HILIC LC Column (50 × 2.1 mm, 2.6 μm) (Thermo Fisher Scientific, Waltham, MA, USA) was used with a mobile phase of solvent A (99% water with 0.1% formic acid) and solvent B (99% acetonitrile with 0.1% formic acid). The column temperature was set at 40 °C. The injection volume was 5 μL. Mass Spectrometer detector QE HF-X (Thermo Fisher Scientific, Waltham, MA, USA) was used to detect the metabolites. The gradient elution program was as follows: (0~0.5 min, 1% B; 0.5~3.5 min, 1~53% B; 3.5~7.5 min, 53~70% B; 7.5~9 min, 70~90% B, 9~13 min, 90% B, 13~14 min, 90~1% B, 14~16 min, 1% B). To ensure the stability and repeatability of operation systems, pooled QC samples were analyzed every 5 runs.

The mass spectrometry mode uses an electrospray ionization source (ESI) that can operate in both positive and negative ion modes. The optimal conditions were set as follows: spray voltage 3.5 kV(+)/2.5 kV(−); transfer tube set at 320 °C; auxiliary gas flow rate 10 arb; scanning mode, Full MS/Data Dependent Secondary Scanning (Full MS/dd-MS2); full MS resolution was 70,000 and MS/MS resolution was 17,500; the detection was carried out over a mass range of 100–1500 *m*/*z*; collision energy gradients of 20, 40, and 60 eV. The instrument was operated using XCalibur 3.0.63 software (Thermo Fisher Scientific Inc., Waltham, MA, USA). The data were imported into Compound Discoverer 3.0 to perform the metabolic feature extraction by the adoption of a molecular feature extraction algorithm (Thermo Fisher, Inc., Santa Clara, CA, USA). The peaks were extracted with parameters as follows: mass deviation, 5 ppm; signal strength deviation, 30%; signal-to-noise ratio, 3; minimal signal strength, 100,000. The possible molecular formula was fitted by extracting the molecular ion chromatographic peak and isotope peak. At the same time, the metabolites were identified by matching the exact *m*/*z* value in online databases (such as mzCloud (https://www.mzcloud.org/, accessed on 7 April 2023), Metlin (https://metlin.scripps.edu/, accessed on 7 April 2023), Human Metabolome Database (http://www.hmdb.ca, accessed on 7 April 2023), and off-line mzVault (Thermo Fisher Scientific, Waltham, MA, USA) using MS/MS fragmentation patterns. The filtering parameters were set as follows: peak area threshold 80,000, primary and secondary quality deviation 5 ppm, and matching score higher than 80. The filtered ions were compared with the compound information in the database to analyze and identify the compounds.

### 2.9. Statistical Analysis

All measurements were presented as mean ± SD. Statistical significance of the differences among different groups was determined by Tukey’s multiple-comparisons test using SPSS (Version 26.0, SPSS Inc., Chicago, IL, USA). A significant difference was set as * *p* < 0.05, ** *p* < 0.01, and *** *p* < 0.001 for comparison with the MD group.

For the 16S rDNA dataset, principal coordinate analysis (PCoA) and clustering heatmap were used to present the variation of microbiome from different groups. Linear discriminant analysis of effect size (LEfSe), non-parametric paired *t*-tests (*p* < 0.05), and fold-change (FC > 1.5) methods were used to analyze changes in differential microbial abundance due to the FEPE intervention. 

For the metabolomics dataset, principal component analysis (PCA), partial least squares discriminant analysis (PLS-DA), and clustering heatmap were used to visualize variation and screen the differential metabolites among different groups. Metaboanalyst 6.0 (https://www.metaboanalyst.ca/, accessed on 8 June 2023) was used to annotate the KEGG pathways of differential metabolites.

## 3. Results

### 3.1. Main Polyphenolic Component Analysis in FEPE Material 

**Figure 2:**
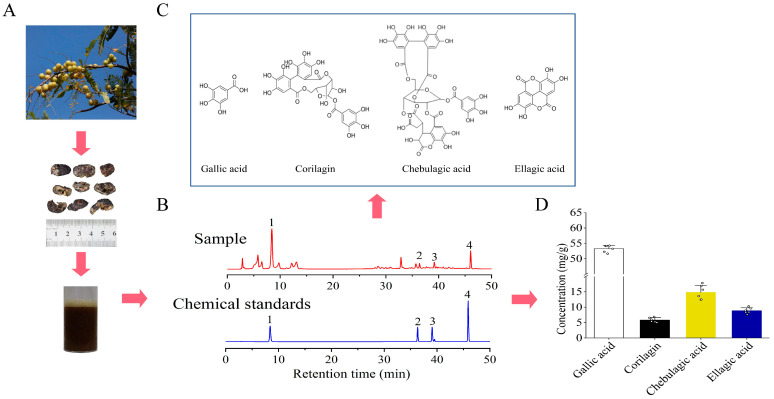
Preparation and representative HPLC-UV chromatogram of the fruit of *Phyllanthus emblica* L. (FEPE) materials. (**A**) Preparation of FEPE materials. (**B**) The chromatographic fingerprints of chemical standards and samples. (**C**) The main chemical constitutes of FEPE. (**D**) Concentration diagram of the main chemical components in FEPE.

We initially analyzed the polyphenolic components in the extract of FEPE using the HPLC technique according to the Chinese pharmacopeia method (Figure 2A) [20]. The standard concentration curves were established by drawing peak areas, and methodological checks such as precision, stability, reproducibility, and recovery were carried out separately (Tables S2 and S3). As seen in Figure 2B, all compounds can achieve an excellent baseline separation and meet the requirements of quantitative analysis.

Comparatively, we observed that the FEPE extract was rich in polyphenolic components. The concentrations of gallic acid, corilagin, chebulagic acid, ellagic acid, and quercetin in FEPE were identified and determined using the external reference method (Figure 2C). Among them, gallic acid was identified as the highest accumulated polyphenolic component in used FEPE extraction (53.29 ± 1.11 mg/g) (Figure 2D). The concentrations of other polyphenolic compounds for corilagin, chebulagic acid, and ellagic acid in the used FEPE materials were 5.85 ± 0.75 mg/g, 14.82 ± 2.30 mg/g, and 8.84 ± 0.97 mg/g, respectively. According to the published studies, these polyphenolic compounds in FEPE may be the foundation for their anti-hyperlipidemia function [12,13,24,25].

### 3.2. Effect of FEPE on HFD-Induced Hyperlipidemia in Mice

**Figure 3:**
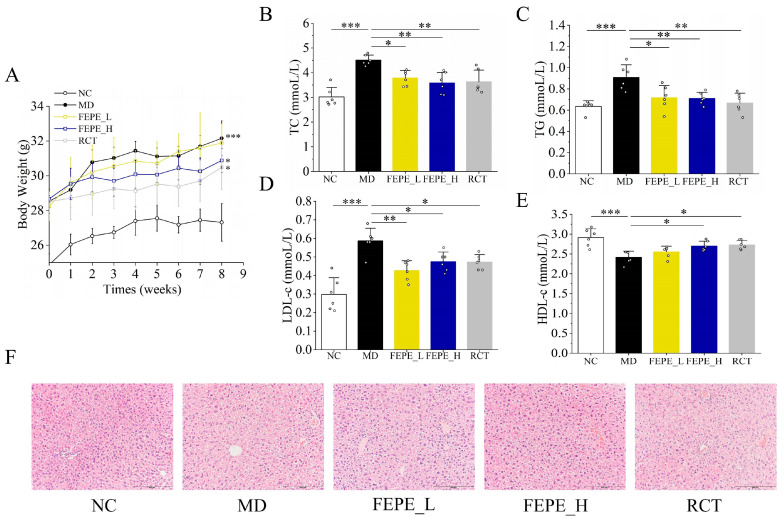
The effect of the fruit of *Phyllanthus emblica* L. (FEPE) on high-fat diet (HFD)—induced hyperlipidemia in mice. (**A**) Body weight change; (**B**) total cholesterol (TC) change level; (**C**) triglyceride (TG) change level; (**D**) low-density lipoprotein cholesterol (LDL-c) change level; (**E**) high-density lipoprotein cholesterol (HDL-c) change level; (**F**) images of H&E-stained liver (200×, scale bar: 200 μm). * *p* < 0.05, ** *p* < 0.01, *** *p* < 0.001.

To examine the ameliorative effect of FEPE on hyperlipidemia, we examined the changes in body weight, plasma lipid levels, and pathological sections of liver tissue in different groups. The results showed that the body weight of mice in the MD group was significantly increased compared to the NC group (*p* < 0.001). The FEPE_H and RCT groups had similar effects in reducing the body weight of mice by 3.97% and 5.21%, respectively (*p* < 0.05, Figure 3A). The levels of TC, TG, and LDL-c in the MD group were remarkably higher than those in the NC group, which was consistent with the trend of body weight. After 8 weeks of intervention, the TC levels in the FEPE_L and FEPE_H groups were reduced significantly by 15.96% and 20.62%, respectively (*p* < 0.05, Figure 3B). The levels of TG in the FEPE_L and FEPE_H groups were significantly reduced by 20.88% and 21.98%, respectively (*p* < 0.05, Figure 3C). FEPE intervention of both low and high doses also significantly decreased the concentration of LDL-c level in blood plasma (*p* < 0.05, Figure 3D) compared with the MD group. About HDL-c levels, there was a significant rise of 11.57% after the FEPE_H intervention. There was an upward trend of 5.79% with the FEPE_L intervention, although it was not statistically significant (Figure 3E). Additionally, the positive drug RCT intervention significantly reduced the concentrations of TC, TG, and LDL-c and slightly increased the concentration of HDL-c. 

Pathological features of liver H&E staining showed that the hepatocytes in the MD group were disordered (Figure 3F). The cytoplasm was filled with fat droplets, and some of the nuclei were associated with typical steatosis. The hepatocytes in the FEPE_L, FEPE_H, and RCT groups had smaller lipid droplets. The cells were arranged in a slightly regular order, and the cell gaps were visible. In summary, the FEPE interventions, especially in high doses, produced an effective alleviating function on HFD-induced hyperlipidemia by significantly reducing the levels of body weight, TC, TG, and LDL-c concentrations and improving the HFD-induced pathological injury of the liver.

### 3.3. Effects of FEPE on Gut Microbiota in Mice

The gut microbiota is recognized to have an essential role in the development of metabolic diseases such as hyperlipidemia, diabetes, and obesity [26,27,28,29]. Multiple studies have demonstrated that polyphenols could effectively regulate the gut microbiota [30,31]. To study the role of gut microbiota in the lipid-lowering effect, we focused on the feces samples from NC, MD, and FEPE_H groups to further analyze the change of gut microbiota after 8 weeks of FEPE intervention. As shown in Figure S1, with the increasing number of sequences, the observed calibration of Sobs and Shannon curves of OTUs leveled off, indicating that the quality and diversity of the sequenced gut microbiota were favorable. 

**Figure 4:**
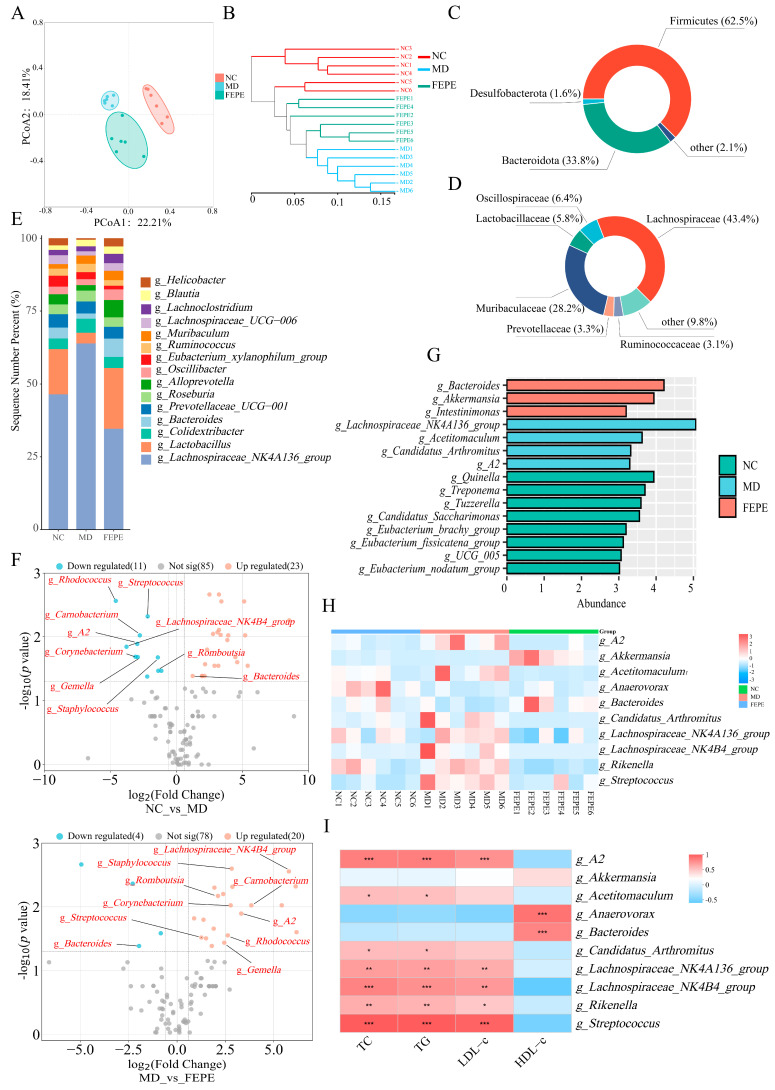
FEPE (the fruit of *Phyllanthus emblica* L.) reshapes the gut microbiota profiles and alters specific microbial taxa in hyperlipidemia mice. (**A**) β-diversity analysis. (**B**) Tree clustering diagram. (**C**) Abundance in phylum level, (**D**) abundance in family level, (**E**) abundance in genus level, (**F**) volcano plot of genus level, (**G**) LEfSe analysis, (**H**) hierarchical clustering heatmap. (**I**) Correlation of differential bacteria and biochemical indices (* *p* < 0.05, ** *p* < 0.01, *** *p* < 0.001).

The PCoA based on weighted Bray-Curtis distance matrices was conducted to present the comprehensive microbial phenotypes among different groups at the OTU level. The results showed that there was a clear separation among the three groups of samples. On the PCoA1 coordinates, it could be seen that the samples in the FEPE group were between the MD and NC groups, with a tendency to move closer to the NC group (Figure 4A). The result from the tree clustering diagram was consistent with that from PCoA (Figure 4B). These results suggested that the 8-week FEPE intervention reversed the alteration of the gut microbial profile caused by a long-term HFD.

Then, the relative abundance of microbial compositions among different groups was analyzed at the taxonomic levels. The gut microbiota in different groups was dominated by the phyla of Firmicutes (62.5%) and Bacteroidetes (33.8%) (Figure 4C). At the family level, Lachnospiraceae (43.4%), Muribaculaceae (28.2%), Oscillospiraceae (6.4%), Lactobacillaceae (5.8%), Prevotellaceae (3.3%), and Ruminococcaceae (3.1%) were identified as core bacterial family due to their relatively high abundances (Figure 4D). Subsequently, at the genus level, we identified significant structural compositional changes among three groups (Figure 4E). The *t*-test (*p* < 0.05) and FC > 1.5 methods were used to search for significantly different bacteria. As shown in Figure 4F, the volcano plot showed that the relative abundances of eleven bacterial genera were significantly downregulated, while those of 23 bacterial genera were significantly up-regulated after the HFD intervention. The intervention of high-dose FEPE significantly reduced the relative abundance of four bacterial genera and significantly increased the relative abundance of 20 bacterial genera. We further analyzed the association between these microbial taxa abundance using the LEfSe method (Figure 4G) with an LDA score > 3 and by one-way analysis of variance (ANOVA) followed by Tukey’s multiple-comparisons tests (*p* < 0.05). We found that the FEPE intervention effectively reversed the abundance of ten key microbes. The high-dose FEPE intervention reversed the significant increase in the relative abundance of *A2*, *Acetitomaculum*, *Candidatus_Arthromitu*s, *Lachnospiraceae_NK4A136_group*, *Lachnospiraceae_NK4B4_group*, *Rikenella*, and *Streptococcus*, and significantly decreased the relative abundance of *Akkermansia*, *Anaerovorax*, and *Bacteroides* caused by a long term HFD (Figure 4H).

Subsequently, we performed Spearman’s correlation analysis between the 10 key bacteria and lipid levels (Figure 4I). The relative abundance levels of *Akkermansia*, *Anaerovorax*, and *Bacteroides* were significantly negatively correlated with TC, TG, and LDL-c levels and significantly negatively correlated with HDL-c levels. The other seven key bacteria were exactly the opposite of that. These results indicated that the 8-week high-dose FEPE intervention might affect lipid levels by influencing the abundance of the key gut microbes.

### 3.4. The FEPE Intervention Ameliorated the Dramatic Reduction in Levels of SCFAs Caused by the Chronic HFD

Accumulating evidence supports the important role of SCFAs, metabolites of the gut microbiota, in the regulation of energy metabolism and lipid levels [32,33]. To elucidate the effects of the high-dose FEPE intervention on SCFAs resulting from long-term HFD, the concentrations of acetic acid, propionic acid, butyric acid, isobutyric acid, valeric acid, and isovaleric acid in mouse feces were determined by the GC-MS method. The calibration curve was successively constructed for each fatty acid compound using the reference standard (Table S4), and their chromatograms were presented in Figure S2. As seen in this figure, the peaks of all the objective compounds were baseline-separated, meaning that this condition could be used for quantitative analysis.

**Figure 5:**
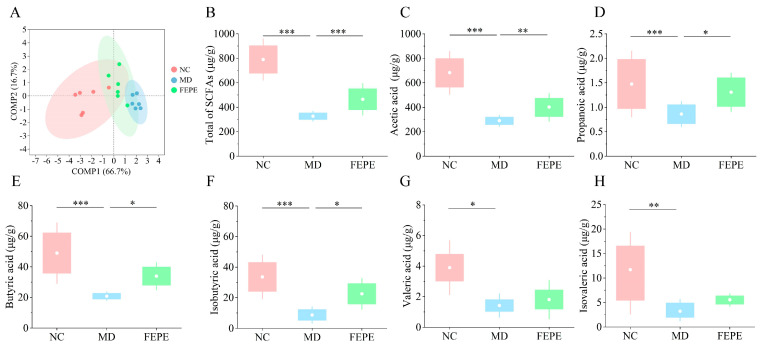
Measurement of short-chain fatty acids (SCFAs) in feces by GC-MS. (**A**) Principal component analysis (PCA) of SCFAs. (**B**) Total of SCFAs. (**C**) Acetic acid. (**D**) Propanoic acid. (**E**) Butyric acid. (**F**) Isobutyric acid. (**G**) Valeric acid. (**H**) Isovaleric acid. * *p* < 0.05, ** *p* < 0.01, *** *p* < 0.001.

We initially constructed a two-dimensional plot based on PCA to overall visualize the differences in the levels of SCFAs among different groups (Figure 5A). The results showed that a long-term HFD affected the changes of SCFAs in mice, especially the MD group, which had a significant trend of separation from the NC group. At the PC1 level, we could see that the FEPE group was traveling away from the MD group and closer to the NC group. The data for the total of SCFAs were consistent with PCA, that a long-term HFD induction significantly reduced the level of SCFAs (*p* < 0.001), and the FEPE intervention reversed this trend (*p* < 0.001) (Figure 5B). Among them, the 8-week FEPE intervention could significantly increase the levels of acetic acid (*p* < 0.01), propionic acid (*p* < 0.05), butyric acid (*p* < 0.05), and isobutyric acid (*p* < 0.05). The high-dose FEPE intervention could increase the levels of valeric acid and isovaleric acid, but there were no significant changes (Figure 5C–H). Acetic acid was the richest in SCFAs in the intestine, which helps to down-regulate the expression of fat-producing related factors such as ACC (Acetyl-CoA carboxylase). Butyric acid and isobutyric acid had a positive effect on cholesterol metabolism [34,35,36]. According to previous studies, FEPE might significantly improve the ability of the gut microbiota to produce SCFAs through the activity of key synthetic enzymes and fortification of genes [37,38]. These results suggested that the high-dose FEPE intervention could reverse the significant level reduction of SCFAs caused by a long-term HFD. 

### 3.5. Metabolomics Analysis

To assess the effect of the FEPE on the metabolome, we performed untargeted metabolomic profiling to quantify the overall composition of the liver metabolites [39,40]. After peak alignment, peak picking, and deconvolution, we detected a total of 18,818 precursor *m*/*z* values in both positive and negative ion modes, respectively. For the quality assessment, the features with RSD values > 30% in the QC samples were removed from all the test samples. Furthermore, unsupervised PCA and the representative total ion chromatograms (TICs) indicated that QC samples were clustered into one category, demonstrating the high stability of the instrument and the repeatability of the method (Figure S3).

**Figure 6:**
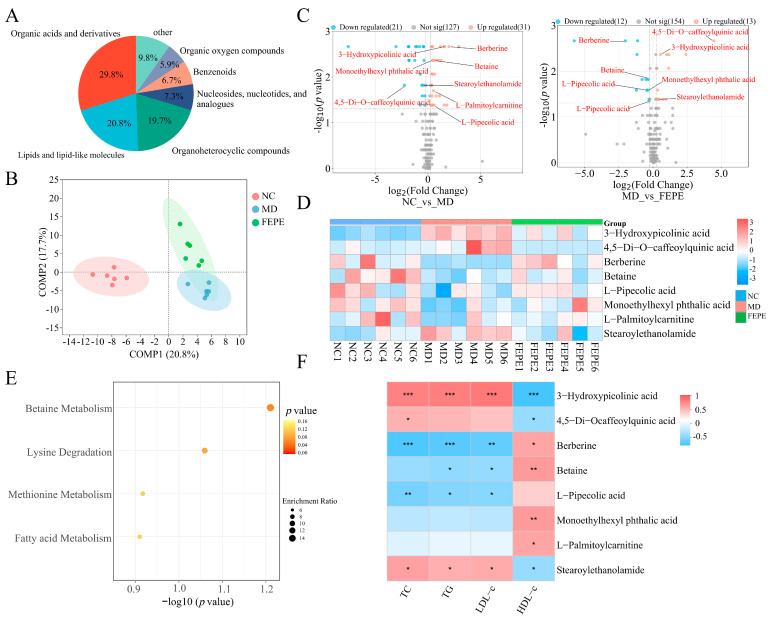
FEPE (the fruit of *Phyllanthus emblica* L.) affects the liver metabolome and metabolic pathway. (**A**) Metabolite type, (**B**) partial least squares discriminant analysis (PLS-DA) model, (**C**) volcano plot, (**D**) hierarchical clustering heatmap, (**E**) metabolic pathway. (**F**) Correlation of differential metabolites and biochemical indices.* *p* < 0.05, ** *p* < 0.01, *** *p* < 0.001.

A total of 755 metabolite features in positive and negative ion modes were reserved, and 178 metabolites were identified based on the annotations available from online databases (HMDB or KEGG). These metabolites primarily belonged to organic acids and derivatives (29.8%), lipids and lipid-like molecules (20.8%), and organoheterocyclic compounds (19.7%) (Figure 6A). The PLS-DA model represented the total variance of 38.5% from PC1 and PC2, exported the parameters of R2 and Q2 of 0.997 and 0.896, respectively, indicating that this model was reliable. As shown in Figure 6B, the PLS-DA model separated the metabolic profiles between different groups well. The volcano diagram was used to identify the metabolites that had a significant effect on the differences between different groups based on the *t*-test (*p* < 0.05) and fold-change (FC > 1.5) methods. The relative abundance of 21 metabolites was significantly down-regulated, and the relative abundance of 31 metabolites was significantly up-regulated due to long-term HFD. The FEPE intervention significantly down-regulated the relative abundance of 12 metabolites and significantly up-regulated the relative abundance of 13 metabolites compared with those in the MD group (Figure 6C). We found that the FEPE intervention effectively reversed the abundance of eight key metabolites. We further exploited these differential features to construct a hierarchical clustering heat map, and the cluster analyses of these eight key differential metabolites showed a clear separation in the heat map (Figure 6D). To determine the key metabolic pathways impacted by the FEPE intervention, we performed an annotation of these key metabolites based on the metaboanalyst 6.0. The main enrichment is in four metabolic pathways, including betaine metabolism, lysine degradation, methionine metabolism, and fatty acid metabolism, and all of them were slightly associated with lipolysis (Figure 6E). The betaine, which acts as a methyl donor, has been found to have hepatoprotective effects and can effectively treat fatty liver disease and metabolic syndrome while inhibiting fat accumulation in the body [41,42]. The metabolic pathway of lysine, one of the essential amino acids in the human body, was related to fatty acid synthesis, fat oxidation, and obesity. Moreover, L-carnitine was an endogenous molecule involved in fatty acid metabolism, and the synthesis of which in the body was closely related to L-lysine and L-methionine [43,44,45].

Subsequently, we performed Spearman’s correlation analysis between these key metabolites and lipid levels (Figure 6F). The relative levels of 3-Hydroxypicolinic acid, 4,5-Di-O-caffeoylquinic acid, and stearoylethanolamide were significantly positively correlated with TC, TG, and LDL-c levels and significantly negatively correlated with HDL-c levels. The other five key metabolites were exactly the opposite of that. These studies illustrated that high-dose FEPE might ameliorate hyperlipidemia by integrally modulating multiple lipid-related metabolic pathways.

### 3.6. Correlation Analysis of Lipid Levels, Key Gut Bacteria, and Key Metabolites

**Figure 7:**
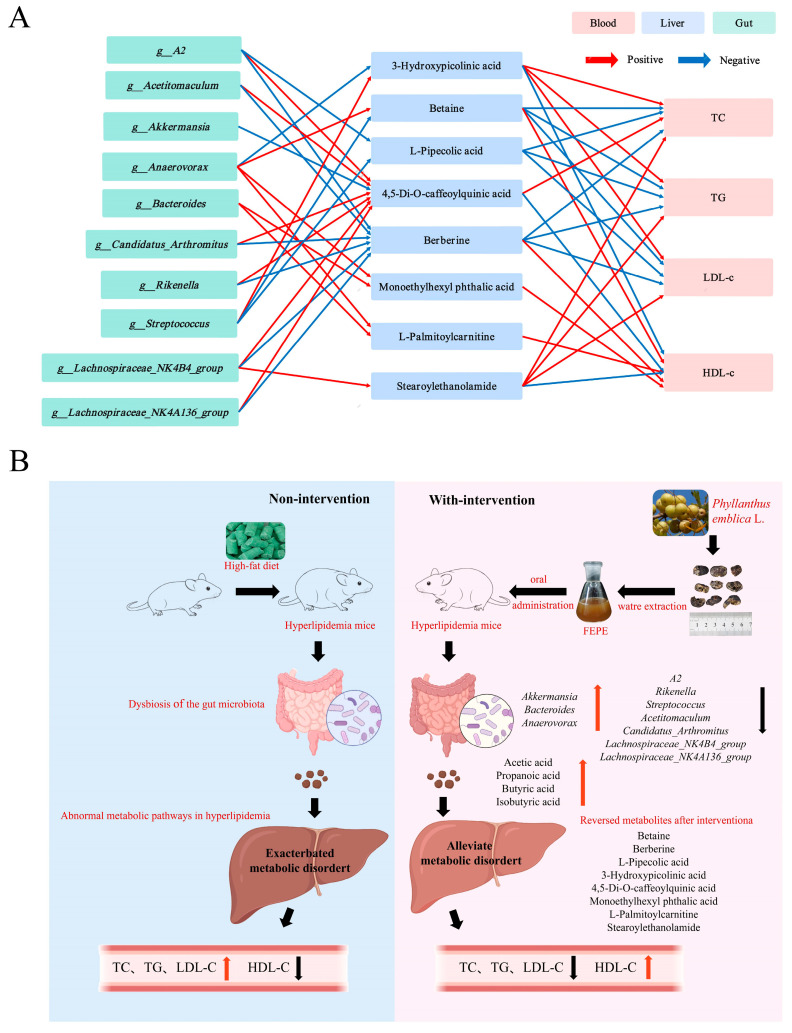
Analysis of possible mechanisms of FEPE (the fruit of *Phyllanthus emblica* L.) intervention in hyperlipidemia. (**A**) Correlation analyses among key bacteria, key metabolites, and lipid levels. (**B**) The specific mechanisms of FEPE intervention in hyperlipidemia.

In light of the biological association among the gut microbiome, gut bacteria, and liver metabolome, correlation analyses were conducted to explore the potential of FEPE extract in treating hyperlipidemia. For the interaction between the gut microbiota and liver metabolome, a total of 24 correlations, including 3 strong correlations (|r| = 0.7–1.0), 13 intermediate-strength correlations (|r| = 0.6–0.7), and 8 weak correlations (|r| = 0.5–0.6), were presented (Figure S4). Out of all the interactions, *g_Lachnospiraceae_NK4B4_group* showed the most significant change, indicating that it might be one of the gut microbiota most affected by the FEPE intervention. 4,5-Di-O -caffeoylquinic acid showed the most altered association with various gut microbiota, suggesting that it might be one of the most affected metabolites by the FEPE intervention (Figure 7A). Combind with the results of correlation analyses of liver tissue metabolite and lipid levels, *Lachnospiraceae_NK4B4_group*-Berberine-TC, TG, and LDL-c and *Lachnosp_raceae_NK4B4_group*-4,5-Di-O-caffeoylquinic acid-TC can be considered as key “microbial metabolite” axes. These results confirmed the interplay of the gut microbiome with the liver metabolome and suggested that the influence of FEPE intervention on the microbiome-liver metabolome axis was the potential mechanism for treating hyperlipidemia.

In summary, we speculated that FEPE might ameliorate hyperlipidemia by modulating the microbiome-liver metabolome axis. As shown in the figure, the FEPE was administered orally to mice to modulate the structural composition of microorganisms, such as increasing the abundance of “good bacteria” (e.g., *g_Akkermansia*, *g_Bacteroides*, *g_Anaerovorax*) and decreasing the abundance of “bad bacteria” (e.g., *g_A2*, *g_Acetitomaculum*, *g_Candidatus_Arthromitus*, *g_Lachnospiraceae_NK4A136_group*, *g_Lachnospiraceae_NK4B4_group*, *g_Streptococcus*, and *g_Rikenella*). At the same time, the content of acetic acid, propionic acid, butyric acid, and isobutyric acid increased. Then, the changes in gut microbes and their metabolites have altered the liver metabolites, which ultimately regulate lipid levels through the metabolic pathways of betaine metabolism, lysine degradation, methionine metabolism, and fatty acid metabolism (Figure 7B).

## 4. Discussion

The extraction of natural products is one of the most important sources of medicinally active ingredients for maintaining human health and plays a vital role in preventing and treating chronic diseases. *Phyllanthus emblica* L. can raise body fluids and alleviate indigestion, according to the Chinese Pharmacopoeia [20]. Furthermore, previous studies have shown that FEPE is a medicinal and food-based material and is considered to be an effective protective agent against metabolic diseases with no adverse effects on human consumption [7,8]. Previous research on FEPE has been limited to weight loss and lipid-lowering effects. However, the exact mechanism of how FEPE affects lipid levels is unknown. In this study, we investigated the effects of FEPE (200 mg/kg) on lipid levels, gut microbiota, and hepatic metabolome in hyperlipidemic mice and their potential associations. These findings may provide valuable insights into the lipid-lowering mechanism of FEPE.

Disorders of lipid metabolism are one of the important signs of hyperlipidemia [1,2]. Our results showed that MD had faster weight gain and higher levels of TC, TG, and LDL-c compared to the NC group, which is consistent with the description above. Notably, the reversal of these changes was evident after the FEPE (100 mg/kg, 200 mg/kg) intervention. The liver tissue sections showed a reduction in hepatocyte vesicular fatdroplets and varying degrees of improvement in cellular sequestration andapoptosis, suggesting that FEPE also has a protective effect on the liver. 

In modern studies, gut microbiota has been strongly correlated with hyperlipidemia. The long-term dietary variations will lead to alterations in the composition of the gut microbiota. Consequently, 16S rDNA high-throughput sequencing was used to analyze the bacterial V3–V4 region in the contents of the mouse cecum to better understand the mechanisms by which FEPE (200 mg/kg) intervenes in hyperlipidemia. Our study agrees with previous research that a prolonged high-fat diet causes changes in the gut microbiome structure. After the intervention by FEPE, this situation improved significantly. The PCoA and clustering tree results validated this conclusion (Figure 4A,B). We have identified 10 key bacteria at the genus level that could serve as important markers of hyperlipidemia induced by a HFD. Among them, *g_Akkermansia*, *g_Lachnospiraceae_NK4A136_group*, and *g_Lachnospiraceae_NK4B4_group* were in agreement with the previous studies, and all of them had a strong correlation with markers related to hyperlipidemia [46,47]. The gut microbes are critical in maintaining cardiovascular health, and if dysregulated, can lead to atherosclerosis, high cholesterol, and hyperlipidemia diseases. *g_Akkermansia,* which is a probiotic, has been particularly useful in lowering sugar and weight loss. A decrease in *g_Akkermansia* could disrupt the intestinal barrier function, leading to increased levels of endotoxins in the blood, ultimately causing low-grade inflammation and metabolic disorders [48,49]. *g_Akkermansia*, prevalent in the FEPE group and scarce in the MD group, emerged as a key probiotic species. One study found that supplementing with *Lactobacillus acidophilus*, a probiotic, can decrease insulin resistance and improve obesity and hyperlipidemia [15,50]. These studies suggested that FEPE might have an anti-hyperlipidemic effect by beneficial gut microbial modulation.

Metabolites of the gut microbiota could also act as signaling molecules to participate in signal transduction and regulate the metabolism of the host [51]. Small molecules of intestinal microbial metabolites can penetrate the intestinal wall and enter the bloodstream, where they are transported through the circulation to various organs, thereby affecting tissue metabolism such as endotoxins, secondary bile acids, and SCFAs [51,52]. Modern studies have demonstrated that the SCFAs were the key signaling molecules participating in the crosstalk between the gut and the liver. Yang et al. showed that long-term administration of gallic acid restored gut microbial imbalances and promoted the secretion of SCFAs [25]. Our research findings are in agreement with theirs; levels of acetic acid, propanoic acid, butyric acid, and isobutyric acid were significantly higher in the high-dose FEPE group compared to the MD group (*p* < 0.05). Previous studies have shown that acetic acid, butyric acid, and isovaleric acid all have positive intervention effects on hyperlipidemia. Acetic acid regulates the expression of Adenosine 5′-monophosphate-activated protein kinase (AMPK) and inhibits the expression of fatty acid synthase in liver tissue [53]. Also, butyric acid can bind to the G-protein-coupled receptor (GPCR) GPR43, which inhibits the breakdown of fatty acids and promotes the secretion of the best-known adiponectin hormone leptin [51]. In summary, FEPE administration orally increased the levels of SCFAs, which could be one of the mechanisms by which FEPE improves hyperlipidemia.

The technology of metabolomics could be used to identify metabolites that differ between hyperlipidaemic patients and healthy individuals, as well as the changes in metabolites after pharmacological interventions. We used a high-resolution UPLC-MS to capture the metabolomic profiles of different groups to analyze the liver metabolome. This technique provided excellent performance for non-targeted metabolite screening. This would help analyze the pathogenesis of hyperlipidemia and the mechanism of action of drugs. For example, a metabolomics study found that 12 biomarkers of metabolic disorders showed a trend of regression after treatment with Wuwei Qingzhuo San, suggesting that hyperlipidemia can be improved by improving metabolic disorders [54]. Our study identified eight key metabolites that could serve as potential markers closely associated with hyperlipidemia, including 3-Hydroxypicolinic acid, 4,5-Di-O-caffeoylquinic acid, berberine, betaine, L-Pipecolic acid, Monoethylhexyl phthalic acid, L-Palmitoylcarnitine, and Stearoylethanolamide. These metabolites were mainly enriched in four metabolic pathways: betaine metabolism, lysine degradation, methionine metabolism, and fatty acid metabolism. All of these pathways have been associated with lipolysis. Betaine is a methyl donor that could effectively inhibit genes related to lipid metabolism and reduce lipid deposition in the liver. Mechanistically, betaine can inhibit nuclear factor-κB activity and NLRP3 inflammasome activation, regulate energy metabolism, and mitigate endoplasmic reticulum stress and apoptosis [47,55,56]. As a result, betaine has positive effects on various human diseases, including obesity, diabetes, cancer, and Alzheimer’s disease. L-Palmitoylcarnitine is a crucial endogenous fatty acid metabolite that breaks down lipids and provides energy to the body. The intervention of FEPE has been found to increase the levels of betaine and L-Palmitoylcarnitine, which could be one of the significant mechanisms to improve hyperlipidemia through betaine metabolism and the fatty acid metabolic pathway.

In the combined analysis of gut microbiota and liver tissue metabolites, *g_Lachnospiraceae_NK4B4_group* was the microbiota with the most significant metabolite-related changes. Consistent with previous findings, the *g_Lachnospiraceae_NK4B4_group* had a strong association with hyperlipidemia [46]. In our study, *g_Lachnospiraceae_NK4B4_group* was positively correlated with 4,5-Di-O-caffeoylquinic acid and berberine and negatively correlated with Stearoylethanolamide. Considering the strength of association (|r| > 0.7), 4,5-Di-O-caffeoylquinic acid and berberine were considered key differential metabolites. 4,5-Di-O-caffeoylquinic acid was positively associated with six gut microbes and negatively associated with one. Berberine was negatively correlated with six gut microorganisms. Combined with the results of correlation analyses of liver tissue metabolite and lipid levels, *Lachnospiraceae_NK4B4_group*-Berberine-TC, TG, LDL-c, and *Lachnospiraceae_NK4B4_group*-4,5-Di-O-caffeoylquinic acid-TC can be considered as key “microbial metabolite” axes. In conclusion, we hypothesize that FEPE might ameliorate hyperlipidemia by modulating the microbiome-liver metabolome axis. The FEPE intervention first changed the structural composition of gut microbiota, such as increasing the abundance of “good bacteria” such as *g_Akkermansia*, *g_Bacteroides*, *g_Anaerovorax*, and decreasing the abundance of “bad bacteria” such as *g_A2*, *g_Acetitomaculum*, *g_Candidatus_Arthromitus*, *g_Lachnospiraceae_NK4A136_group*, *g_Lachnospiraceae_NK4B4_group*, *g_Streptococcus*, and *g_Rikenella* and increasing the level of SCFAs. Secondly, it improves hyperlipidemia by reducing body weight and lipid levels, protecting the liver, and reducing liver damage through betaine metabolism, lysine degradation, methionine metabolism, and fatty acid metabolism pathways. 

Additionally, the primary mechanism through which statins exert their anti-hyperlipidemic effects is the inhibition of HMG-CoA reductase activity. Existing research has shown that key metabolic components in FEPE, such as gallic acid and ellagic acid, both exhibit inhibitory effects on HMG-CoA reductase. For instance, some studies have found that in rats with T2DM) induced by STZ, the activity of HMG-CoA reductase is significantly elevated, and intervention with gallic acid could significantly reduce the activity of HMG-CoA reductase. Supplementation with quercetin and gallic acid can significantly reduce the activity of HMG-CoA reductase in rats fed a high-cholesterol diet [57,58,59,60].

In contrast, the present study primarily investigates the potential mechanisms underlying the therapeutic effects of FEPE against hyperlipidemia from the perspectives of gut microbiota and liver metabolism. The findings may provide a further reference for studying the mechanisms by which FEPE exerts its anti-hyperlipidemic effects. However, the elucidation of more precise mechanisms necessitates additional experimental validation. Secondly, it is essential to determine whether FEPE can exert hypolipidemic effects through its inhibitory activity on HMG-CoA reductase. The outcomes of these experiments will be instrumental in advancing our understanding of FEPE’s therapeutic potential.

## 5. Conclusions

This study used an integrated approach, including lipid levels, histopathology, 16S rDNA sequencing, and metabolomics, to elucidate the efficacy of FEPE in hyperlipidemia.

Research findings indicate that FEPE, a therapeutic agent rich in polyphenols, was effective in reducing body weight and hyperlipidemia-associated dyslipidemia and liver damage. Notable in our findings was the remarkable ability of FEPE to correct gut microbiota dysbiosis and metabolite disorders. In addition, we found that FEPE also increased the secretion of SCFAs, and this might be one of the mechanisms of FEPE to ameliorate hyperlipidemia. We found that FEPE probably affects “*Lachnospiraceae_NK4B4_group*-Berberine-TC, TG, and LDL-c and *Lachnospiraceae_NK4B4_group*-4,5-Di-O-caffeoylquinic acid-TC” to improve hyperlipidemia. This study provided a theoretical basis for the development and application of functional products containing FEPE from the perspective of the microbiome-liver metabolome axis.

## Data Availability

The raw data supporting the conclusions of this article will be made available by the authors on request.

## Supplementary Materials

The following supporting information can be downloaded at: https://www.mdpi.com/article/10.3390/metabo14050257/s1, Figure S1: Sobs and Shannon curves at the OTU level; Figure S2: Short-chain fatty acid content in feces; Figure S3: Three-dimensional principal coordinate analysis (PCA) plot; Figure S4: Correlation analysis between gut microbes and liver metabolites; Table S1: The composition of mouse feed; Table S2: The method validation of developed HPLC-UV method; Table S3: Regression equation, correlation coefficient, linear range, limit of detection (LOD) and limit of quatification(LOQ) of 4 reference substances; Table S4: Regression equation, correlation coefficient, linear range of 6 reference substances.
