# Supplementary material for: Microbiome-Metabolome Analysis Insight into the Effects of the Extract of Phyllanthus emblica L. on High-Fat Diet-Induced Hyperlipidemia
Source: Metabolites. 2024 Apr 29;14(5):257. doi: 10.3390/metabo14050257 (PMC11123125; doi:10.3390/metabo14050257)
Supplement: Supplementary file 1 [file metabolites-14-00257-s001.zip › metabolites-2945533-SI.pdf]

Supplementary data

Table S1. The composition of mouse feed

**D12450B** (10% calories from fat)			**D12451** (45% calories from fat)	
**Energy supply ratio**	**gm%**	**Kcal%**	**gm%**	**Kcal%**
Protein	19.2	20	24	20
Carbohydrate	67.3	70	41	35
Fat	4.3	10	24	45
Kcal/gm	3.85	100	4.73	100
**Ingredient**	**gm**	**Kcal**	**gm**	**Kcal**
Casein, 80 Mesh	200	800	200	800
L-Cystine	3	12	3	12
Corn Starch	315	1260	72.8	291
Maltodextrin 10	35	140	100	400
Sucrose	350	1400	172.8	691
Cellulose, BW200	50	0	50	0
Soybean Oil	25	225	25	225
Lard*	20	180	177.5	1598
Mineral Mix S10026	10	0	10	0
DiCalcium Phosphate	13	0	13	0
Calcium Carbonate	5.5	0	5.5	0
Potassium Citrate, 1	16.5	0	16.5	0
H20	10	40	10	40

Table S2. The method validation of developed HPLC-UV method

Compounds	Precisions(n=6)	Stability(24h,n=6)	Repeatablity(n=6)	Recovery(n=3)	
	RSD%	RSD%	RSD%	Average Recovery(%)	RSD%
Gallic acid	0.5%	0.9%	2.0%	100.0%	2.2%
Corilagin	0.3%	0.8%	1.6%	99.9%	2.5%
Chebulagic acid	0.7%	0.8%	1.9%	101.1%	2.2%
Ellagic acid	0.4%	1.0%	1.7%	100.4%	3.4%

(The HPLC-UV method was validated in terms of precision, stability, repeatability and accuracy. The precision was assessed based on six replicate measurements of the same sample solution in one day. To evaluate stability, the same sample solution was stored at 4°C and analyzed after 0, 2, 4, 8, 12 and 24 h, respectively. Repeatability was evaluated by independent analysis of six different sample solutions prepared from the same sample. Recovery tests were carried out to investigate the accuracy of the HPLC-UV method using the standard addition method. These parameters showed that this HPLC-UV method was effective to quantity the bioactive compounds in used FEPE medicinal materials.)

Table S3 Regression equation, correlation coefficient, linear range, LOD and LOQ of 4 reference substances

Compounds	Calibration curve	Correlation coefficient	Linear range /mg·L^{-1}	LOD/ mg·L^{-1}	LOQ/ mg·L^{-1}
Gallic acid	Y=14 463*X*-70 447	0.999 6	92.24~1 477.00	50.32	167.73
Corilagin	Y=13 158*X*-35 837	1.000 0	8.50~543.75	2.92	9.75
Chebulagic acid	Y=5 626.5*X*-88 455	0.999 8	18.91~605.00	13.51	45.02
Ellagic acid	Y=19 574*X*-3 859.2	0.999 9	30.96~663.60	10.09	33.62

(According to literature survey, four bioactive compounds were determined to characterize the quality of the fruit extract of *Phyllanthus emblica* L. (FEPE) medicinal materials used in our study. For each reference standard, calibration curve was developed by plotting its peak area against the standard concentration. As seen in this table, the correlation coefficients are all higher than 0.999, and all the determination of bioactive compounds are within the linear range. These parameter showed that this HPLC-UV method was effective to quantity the bioactive compounds in used FEPE medicinal materials.)

Table S4 Regression equation, correlation coefficient, linear range of 6 reference substances

Compounds	Calibration curve	Correlation coefficient	Linear range /μg·L^{-1}
Acetic acid	Y=68878x-1E+06	0.9995	30~240
Propionic acid	Y=50017x-49315	0.9993	12~120
Isobutyric acid	Y=108395x+20306	0.9994	1.2~12
Butyric acid	Y=102911-357391	0.9998	20~160
Isovaleric acid	Y=105325x+92973	0.9999	3.2~32
Valeric acid	Y=101145x+176800	1	2~20

The content of six short-chain fatty acids in faeces was determined according to a literature survey. For each reference standard, calibration curve was developed by plotting its peak area against the standard concentration. As seen in this table, the correlation coefficients are all higher than 0.999, and all the determination of bioactive compounds are within the linear range. The results showed that the GC-MS method was effective in determining the content of short-chain fatty acids in faeces.

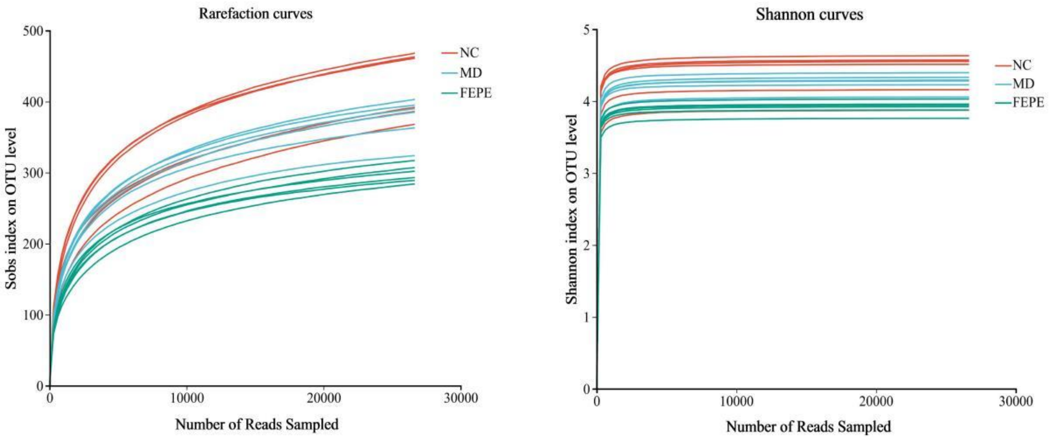

Figure S1 Sobs and Shannon curves at the OTU level

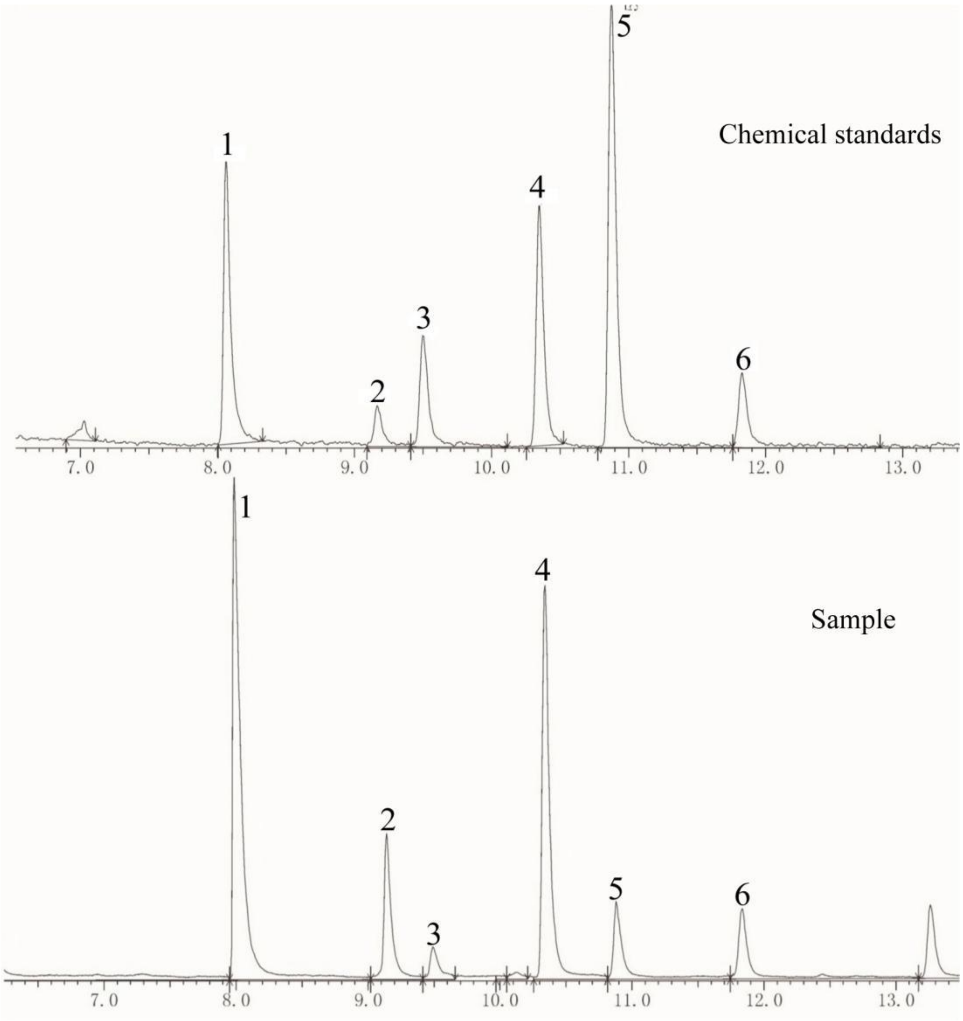

Figure S2 Short-chain fatty acid content in feces. (1) Acetic acid, (2) Propionic acid, (3) Isobutyric acid, (4) Butyric acid, (5) Isovaleric acid, (6) Valeric acid

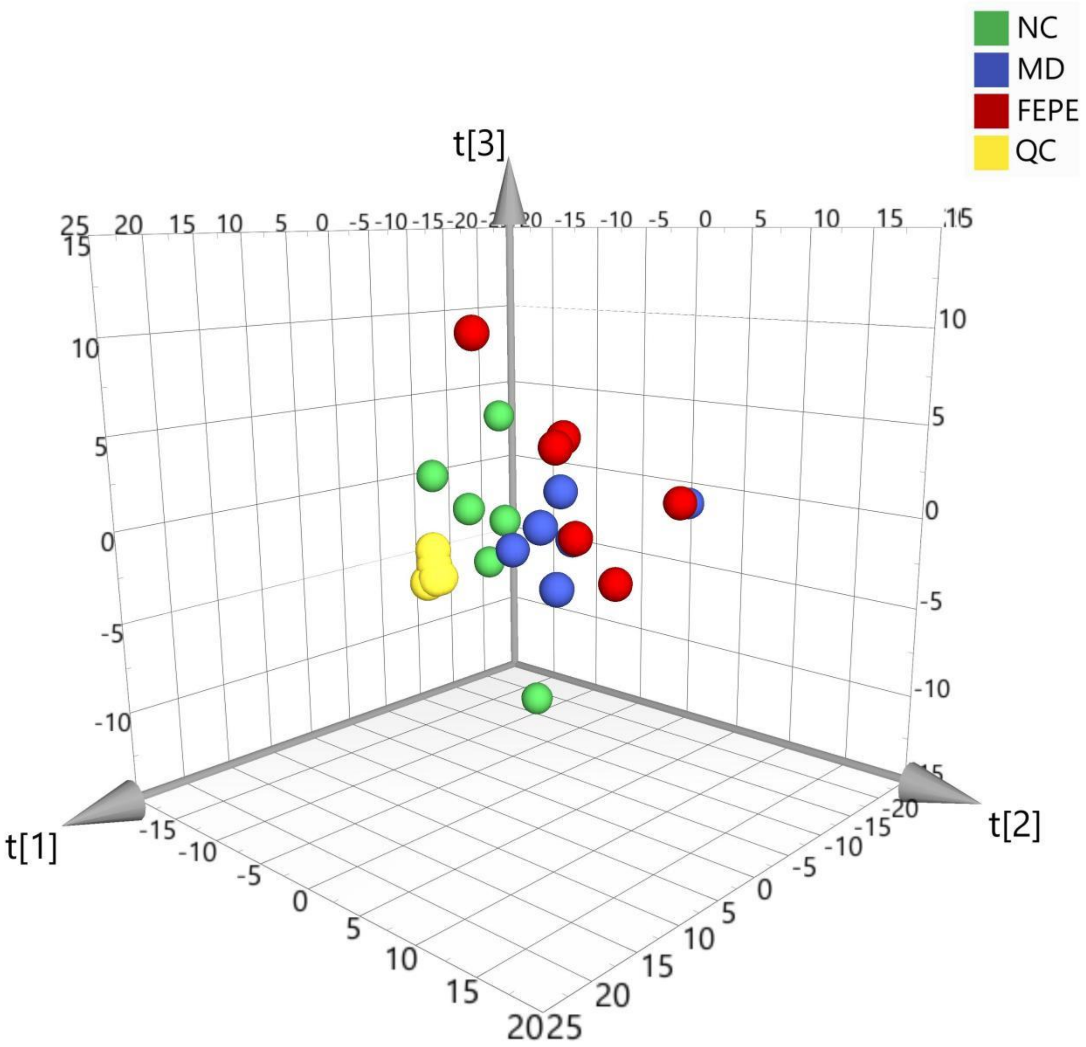

Figure S3 Three-dimensional PCA plot. $R^2(X)$ = 0.514, Q^2 = 0.234; t[1], t[2], and t[3] display the first three principal components.

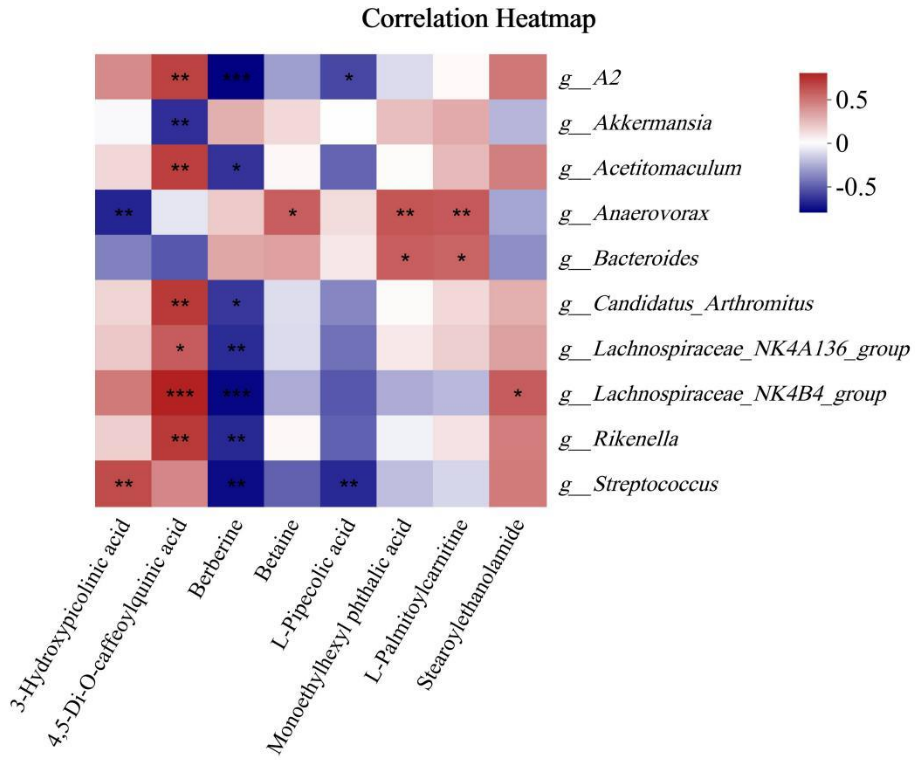

Figure S4 Correlation analysis between gut microbes and liver metabolites.
